# Cross-sectional associations between patterns and composition of upright and stepping events with physical function: insights from The Maastricht Study

**DOI:** 10.1186/s11556-024-00343-w

**Published:** 2024-05-09

**Authors:** Joshua Culverhouse, Melvyn Hillsdon, Annemarie Koster, Hans Bosma, Bastiaan E. de Galan, Hans H.C.M. Savelberg, Richard Pulsford

**Affiliations:** 1 Department of Public Health and Sport Sciences, University of Exeter, Exeter, UK; 2 Department of Social Medicine, Maastricht University, Maastricht, Netherlands; 3 Care and Public Health Research Institute (CAPHRI), Maastricht University, Maastricht, Netherlands; 4 School for Nutrition and Translational Research in Metabolism (NUTRIM), Maastricht University, Maastricht, the Netherlands; 5 Department of Human Movement Sciences, Maastricht University, Maastricht, the Netherlands; 6 Department of Internal Medicine, Maastricht University Medical Centre, Maastricht, the Netherlands; 7 Cardiovascular Research Institute Maastricht (CARIM), Maastricht University, Maastricht, the Netherlands; 8 Department of Internal Medicine, Radboud University Medical Centre, Nijmegen, the Netherlands

**Keywords:** Physical activity, Accelerometer, Patterns, Fragmentation, Posture, Physical function

## Abstract

**Introduction:**

Age-related declines in physical functioning have significant implications for health in later life. Physical activity (PA) volume is associated with physical function, but the importance of the pattern in which PA is accumulated is unclear. This study investigates associations between accelerometer-determined daily PA patterns, including composition and temporal distribution (burstiness) of upright and stepping events, with physical function.

**Methods:**

Data was from participants who wore an activPAL3 accelerometer as part of The Maastricht Study. Exposures included a suite of metrics describing the composition and the temporal distribution (burstiness) of upright and sedentary behaviour. Physical function outcomes included the six-minute walk test (6MWT), timed chair-stand test (TCST), grip strength (GS), and SF-36 physical functioning sub-scale (SF-36pf). Multivariable linear regression models were used to assess associations, adjusting for covariates including overall PA volume (daily step count).

**Results:**

Participants(*n* = 6085) had 6 or 7 days of valid data. Upright and stepping event metrics were associated with physical function outcomes, even after adjusting PA volume. Higher sedentary burstiness was associated with better function (6MWT, TCST, and SF-36pf), as was duration and step volume of stepping events (6MWT, TCST, GS, and SF-36pf), step-weighted cadence (6MWT, TCST, and SF-36pf). Number of stepping events was associated with poorer function (6MWT, GS, and SF-36pf), as was upright event burstiness (SF-36pf). Associations varied according to sex.

**Conclusion:**

Our study reveals that diverse patterns of physical activity accumulation exhibit distinct associations with various measures of physical function, irrespective of the overall volume. Subsequent investigations should employ longitudinal and experimental studies to examine how changing patterns of physical activity may affect physical function, and other health outcomes.

**Supplementary Information:**

The online version contains supplementary material available at 10.1186/s11556-024-00343-w.

## Introduction

Low or declining physical functioning with ageing has important implications for future health outcomes [1–4]. The recognised progression from reduced physical function to frailty and disability [5, 6], results in considerable economic burden [7, 8]. Declines in physical function may occur as early as mid-life [9–13] yet changes in physical function are unlikely to be routinely assessed until later in life when presenting in clinical settings, when already significantly impacting an individual’s ability to perform activities of daily living. The prevalence poor physical function is high in general populations with 20–50% recording slow gait speed, and 20% with weak grip strength [14, 15]. Prevalence is higher with increasing with age, co-morbid health conditions, smoking, and in women [16–18] Consequently, maintenance of function, or retarding functional decline is recognised as a public health priority by the World Health Organisation [19]. 

Physical activity is an established determinant of physical function. Systematic reviews demonstrate strong evidence for the beneficial effect of physical activity and exercise interventions for clinically meaningful changes in physical function [20–22] and prevention of frailty [23]. In addition, positive changes in physical activity have been shown to be associated with increased physical function [24]. Longitudinal observational evidence suggests physical activity is associated with physical function and frailty in older adults [23, 25, 26]. 

Much of the evidence for the benefits of physical activity have been based on self-report measures [21, 25, 26], which are effective for capturing types and domains of physical activity that are planned, structured and sustained for a reasonable duration. However, self-reports are less capable of capturing patterns of physical activity accumulation and physical activity behaviour that is more transient and/or incidental [27, 28], due to biases resulting from recall error [29]. 

Device-based measures of movement overcome many of the limitations of self-reports [29] and have become common place in physical activity research [30, 31]. However, existing research into associations between device based physical activity and physical function largely rely on aggregate measures of physical activity volume [31]. For example, average minutes per day of physical activity recorded above cut-points/thresholds (predetermined acceleration values) when using accelerometers, or average daily step count when using step counting devices/outcome. These aggregate measures ignore potentially important differences in how activity is accumulated, with evidence that the pattern and/or timing of physical activity accumulation may be important even after adjustment for volume [32]. In addition, the reliance on single thresholds of acceleration to define physical activity intensities can lead to misclassification, as this approach assumes that any physical activity above the threshold represents the same intensity for all individuals regardless of fitness [33]. More recent developments in the processing of accelerometer data permit greater insight into the importance of how a given volume of physical activity is accumulated, and different ‘patterns’ of activity are potentially differentially associated with health outcomes, including physical function [32, 34, 35]. 

Physical activity characterised by short, transient events, often labelled as fragmented activity, has been associated with various health outcomes, including physical function, even after adjustment for total volume of physical activity [35–38]. One limitation of much of this evidence, and the wider physical activity field arises from its reliance on epoch-based activity measures, where active events are defined as contiguous minutes registering a specified acceleration or count threshold [37]. The methods used to quantify how fragmented physical activity is segment the data into epochs, typically 60 s in fragmentation studies [35–37, 39], and then classify each epoch as active or inactive based on an average acceleration. This approach could lead to misclassification of what is the start or end of an active event. For instance, in a situation where an individual walks briskly for 10 s and then sits for 50 s, the average acceleration for the minute could be above the threshold to be classified as ‘active’, overlooking the potentially significant interruption in activity [40]. This would lead to an underestimate of the true level of fragmentation and an overestimate of the average duration of physical activity events. Further, most of these studies are restricted to simple classifications of active or inactive epochs and therefore do not explore the composition of the active events.

An alternative approach which offers more detail and precision involves ‘event-based’ analysis that segments the data into a contiguous time-series of postures (sit/lying, standing, ambulating) [41]. A time-series of different postures allows upright and stepping events to be quantified by their composition, and temporal distribution [42, 43]. Very limited evidence exists on the association between event-based physical activity metrics and health outcomes. Palmberg et al. [38] examined the fragmentation of minute-by-minute posture classifications (upright or sit\lying postures) and reported that more fragmented upright time was positively associated with mental fatigue. In addition, event-based analysis allows for the examination of the temporal distribution of these events. Preliminary work has looked at the temporal distribution of physical activity over a short period (90 min), using the inter-event time distribution, or ‘burstiness’ parameter [44]. This metric quantifies how clustered or uniformly distributed events are across a specified time period, which provides potential additive information about temporal patterns that are not included in fragmentation metrics.

To our best knowledge, no studies have explored the composition of upright events and stepping events, or their temporal distribution (burstiness). If patterns of accumulation of physical activity are associated with physical function, independent of volume of physical activity, then there is the potential to broaden the current physical activity guidelines that primarily focus on increasing volume. This study aims to investigate the association between event-based metrics that capture the composition and temporal distribution of upright and stepping events with measures of physical function, including grip strength, the six-minute walk test, chair-rise test, and SF-36 physical functioning score.

## Methods

### Study design and participants

We used data from The Maastricht Study, an observational prospective population-based cohort study. The rationale and methodology have been described previously [45]. In brief, the study focuses on the aetiology, pathophysiology, complications and comorbidities of type 2 diabetes mellitus (T2DM) and is characterized by an extensive phenotyping approach. Eligible for participation were all individuals aged between 40 and 75 years and living in the southern part of the Netherlands. Participants were recruited through mass media campaigns and from the municipal registries and the regional Diabetes Patient Registry via mailings. Recruitment was stratified according to known T2DM status, with an oversampling of individuals with T2DM, for reasons of efficiency. The present report includes cross-sectional data from the first 7689 participants, who completed the baseline survey between November 2010 and December 2017. The examinations of each participant were performed within a time window of three months, including the accelerometer measures described below. The study has been approved by the institutional medical ethical committee (NL31329.068.10) and the Minister of Health, Welfare and Sports of the Netherlands (Permit 131088-105234-PG). All participants gave written informed consent.

### Assessment of physical activity

The monitoring of posture and movement behaviour was conducted using the activPAL3™ accelerometer (PAL Technologies Ltd., Glasgow, UK). The activPAL device has shown high criterion validity across validation studies for characterising posture [46], and stepping activity including step count and stepping time used to calculate step-rate/cadence [47]. Accuracy is reduced for slower paced stepping [48], and activity intensity [47], though intensity outputs are not used in this study. At the end of the examination described above, the device was waterproofed using a nitrile sleeve and then attached to the anterior of the right thigh using transparent 3 M Tegaderm™ tape. The activPAL records acceleration and estimates posture (sitting or lying, standing, and stepping) based on proprietary algorithms. Participants were instructed to wear the device continuously 24 h·d^− 1^ for eight days without removing, and not reapply the device once removed. We re-processed the raw activPAL data files using VANE algorithm in the PALbatch software v.8 and cleaned the stepping output using a previously described process [43]. Briefly, the software’s suggested default minimum upright and non-upright (sedentary) duration of 10-seconds was employed. To isolate valid waking wear time from sleep, waking wear time was estimated using the first upright event ≥ 10 s after 03:00 h until the event preceding the one that crossed the following midnight. This estimation method was based on the average midsleep point reported in a large UK cohort study [49], and assumed that the next upright event ≥ 10 s after this midsleep point represented the arise time. The first day of recording was a partial day and was excluded. A minimum of 10 h of waking wear and > 3 upright events (≥ 10 s) was required for a day to be valid, and inclusion criteria for this study was a minimum of six valid days. The variables used from the stepping output to produce the metrics described below were; date/time, event-type, duration, and step count (for stepping events). Upright events were output as a time series with a date and time stamp for each event. Upright events were defined as the time between two consecutive transitions from a sedentary posture to an upright posture, and the subsequent transition from an upright posture back to a sedentary posture. Upright event metrics (described below) were derived for the waking wear time of each 24-hour period and averaged per person across valid days [39]. 

### Composition of upright and stepping events

The mean daily value of the following metrics was derived per person; step count (steps/day), number of upright events (n/day), number of stepping events (n/day), mean duration of stepping event (min), number of steps per stepping event (steps/event), and mean step-weighted cadence of all stepping events (steps/min). A minimum of ten steps was employed during cadence calculation, as it has been determined that 6 to 10 consecutive steps are necessary to precisely capture stepping cadence [50]. The minimum cadence that activPAL reports is 20 steps/minute. The characteristics of each individual upright event were defined by its duration (mins), the percentage of time spent stepping (%), the count of stepping events (n/event), and the step count (steps/event). The mean values of these four within event composition metrics, across the measurement period, were calculated per person. All metrics are described in Table 1.

**Table 1:** Summary of composition and temporal duration metrics of upright and stepping events

Composition of upright and stepping events	Description	Units
Daily step count	Average number of steps per day across the measurement period. Indicator of volume of physical activity	steps/day
Daily upright events	Average number of upright events per day across the measurement period. Equivalent to the number of sit-to-stand transitions per day	n/day
Daily stepping events	Average number of stepping events per day across the measurement period. Indicator of how fragmented stepping behaviour is across the day	n/day
Duration of stepping events	Average duration of all stepping events across the measurement period. Indicator of capacity	min/event
Steps per stepping event	Average number of steps per stepping event across the measurement period. Indicator of capacity.	steps/event
Step-weighted cadence	Average step-weighted cadence per day across the measurement period. Calculated as the mean daily step-weighted cadence (weighted by steps per event) of all stepping events. Indicator of step-rate (a proxy for intensity) that takes into account all steps	steps/min
Upright event duration	Average duration of all upright eventacross the measurement period	min
Proportion of stepping to standing time	Average proportion of time spent stepping when upright across the measurement period	%
Stepping events per upright event	Average number of stepping events per upright event per day. Indicator of how fragmented stepping is *within* upright events on average	n/event
Steps per upright event	Average number of steps per upright event per day. Indicator of the average stepping volume per upright event	steps/event
**Temporal distribution of upright and stepping events**	**Description**	**Units**
Upright event burstiness	Average daily upright event burstiness (inter-event time distribution) across the measurement period. Indicator of the degree to which upright events are clustered together with longer sedentary events between clusters, versus a more uniform distribution of upright events through the day	*B*_n_
Sedentary event burstiness	Average daily sedentary event burstiness (inter-event time distribution) across the measurement period. Indicator of the degree to which sedentary events are clustered together with longer upright events between clusters, versus a more uniform distribution of sedentary events through the day	*B*_n_

### Temporal distribution of upright and stepping events

The temporal distribution of upright and stepping events was described by the ‘burstiness’ parameter, which is based on inter-event time distribution [51]. The burstiness coefficient can range from − 1 to + 1, with a value of -1 for a uniform time-series of events, 0 for a Poissonian or random time-series, and + 1 for ‘extreme’ standard deviation of inter-event times [52]. Burstiness was calculated per day of waking hours and averaged per person. The following equation was used to compute burstiness correcting for the number of events: [44]

$${B_n} = \frac{{\sqrt {n + 1} \left( {\frac{\sigma }{{\left\langle \tau \right\rangle }}} \right) - \sqrt {n - 1} }}{{(\sqrt {n + 1} - 2)\left( {\frac{\sigma }{{\left\langle \tau \right\rangle }}} \right) + \sqrt {n - 1} }}$$

where n, σ, and 〈τ〉 denote the number of events, the standard deviation of inter-event time, and the mean of inter-event time, respectively. The formula was utilized to compute the inter-event time distribution of both upright events (inter-event time being the duration of sedentary events) and sedentary events (inter-event time being the duration of upright events). A lower *B*_n_ value signifies a smaller standard deviation of inter-event times compared to the mean and thus lower burstiness, whereas a higher *B*_n_ signifies a larger standard compared to the mean and ‘burstier’ behaviour [52]. 

**Fig. 1:**
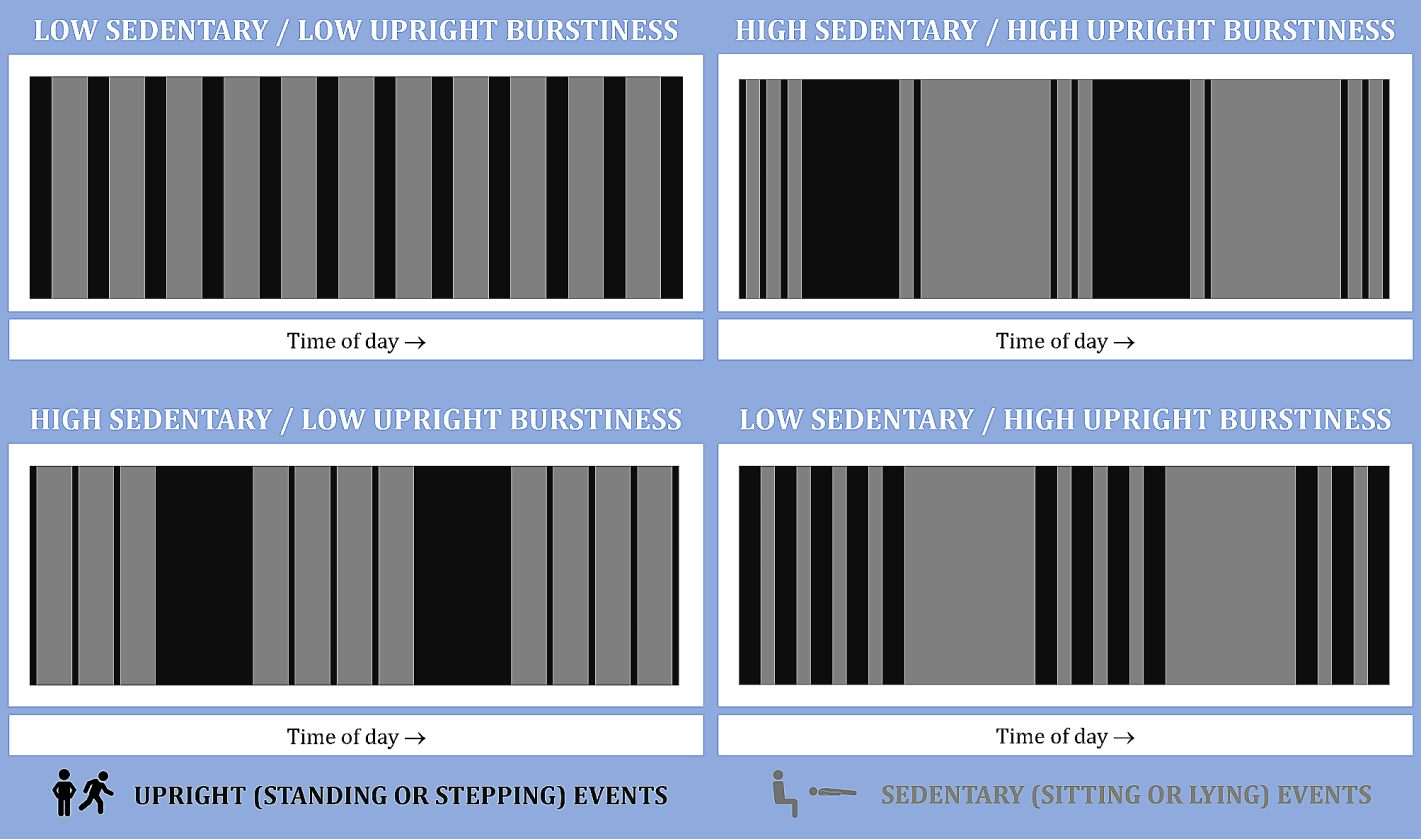
Diagrammatic examples of sedentary and upright event burstiness. The accelerometer wear time and total upright time for each day is matched. Grey bars denote upright events, white bars denote sedentary events. The low burstiness examples would be represented by a burstiness coefficient equal to -1, and the high burstiness examples would be represented by a burstiness coefficient equal to + 0.5 (Reprinted with permission from Culverhouse et al. 2024) [43]

Fig. 1. illustrates the concept of both high and low sedentary and upright event burstiness. To ensure a fair comparison, these examples are matched for the daily number of events, daily waking wear time, and daily duration of upright events (and therefore daily duration of sedentary events). The low/low example shows an even distribution of both event types across the day. The high sedentary / low upright example has an even distribution of upright events (the sedentary events are of consistent duration) but is characterised by two longer durations of upright events, accompanied by a number of short upright events. In other words, high burstiness of sedentary events is only achieved with a mix of longer and shorter duration upright events. In contrast, high upright event burstiness (see low sedentary / high burstiness) is characterised by the clustering of a number of upright events with short gaps between them followed by two much longer periods of being sedentary. The high/high example shows that you can achieve a combination of these, as they are independent (beyond the finite period in which they occur). The examples provided in Fig. 1. are intended to visually demonstrate the concept of burstiness, but in reality, movement data will represent a much more intricate and varied picture.

### Assessment of physical function

Physical function was assessed using three performance-based measures, the protocols for which have been detailed elsewhere [53], and the self-reported Short-Form 36 survey (SF-36) [54]. The SF-36 was self-reported, and the physical function subscale (SF-36pf) was used as the continuous (0-100) outcome variable [55]. The reliability and validity of SF-36pf in older adults has been determined [56]. The protocols for performance-based measures are briefly described below.

### Six-minute walk test

Participants were instructed to walk between two markers, spaced 20 m apart, at a fast pace without running. Standardised encouragement was given every minute. After 6-minutes, or when the participant halted the test, the distance covered was measured. The distance of the six-minute walk test (6MWD) was used as the continuous outcome variable.

### Timed chair stand test

Participants were instructed to stand from a sitting position into a full upright position and sit down again as quickly as possible from a 46 cm high chair with a straight back, no arm-rests, and arms across their chest. The time in seconds to perform 10 repetitions (TCST) was measured to the nearest tenth of a second and used as the continuous outcome variable.

### Handgrip strength

Using the Jamar handheld dynamometer (SEHAN Corp., Korea-Biometrics Europe BV, Almere), participants were instructed in to stand against a wall with the elbow flexed to 90° and squeeze as hard as possible for 3–5 s, while given standard encouragement. Performed three times on each hand, alternating hands between measures, the maximum strength from each trial was recorded (kg). Maximum strength (HGS) overall was used as the continuous outcome variable.

### Covariates

Covariates were selected a priori based on the commonly selected covariates in the literature that are known to influence physical activity, as well as covariates shown to be associated with the upright and stepping metrics within this study [43]. These included age (in years) and sex. Body mass index (BMI) was calculated using the standard formulae (kg)/height (m) [2], using values from measurements taken during the examination. BMI was kept continuous in analyses but reported in the descriptives table using standardised categories of; healthy weight (15 to 24.9 kg/m²), overweight (25 to 29.9 kg/m²), obese (30 to 39.9 kg/m²), and severely obese (≥ 40 kg/m²). Education level was divided into low ((un)completed primary education, or lower vocational education), middle (intermediate vocational education or higher secondary education), and high (higher vocational education or university education). Smoking status was categorised into non-smoker, former smoker, and current smoker. Presence of Type 2 diabetes was defined according to the fasting glucose state and directly after an oral glucose tolerance test and the use of glucose lowering medication (SCHRAM) and was included in the main model as a binary variable. Dutch Healthy Diet index (DHD) score, (which includes assessment of alcohol consumption), was used as measure of diet quality [57]. 

### Statistical analyses

Participant characteristics were described by sex and presented as mean ± SD for continuous variables and number (%) for categorical variables. Multivariable linear regressions were used to assess the variation in upright event metrics across participant characteristics. Further multivariable linear regression models were used to assess the associations of each upright/stepping event metric with each physical function outcome. Associations were expressed as a one standard deviation increase in the upright/stepping event metric equates to an absolute change in the physical function outcome. The associations in model 1 were adjusted for age, sex, and waking wear time. Model 2 was further adjusted for education level, BMI, smoking status, and type 2 diabetes (to account for oversampling in the study). Model 3 was additionally adjusted for daily step count (step volume), to test if the associations persisted over and above a traditional metric of activity volume. Given the established sex-related differences in physical activity [58] and physical function [59, 60], we tested and reported sex interaction effects. Subsequently, for consistency, all analyses were stratified by sex. The interaction with diabetes (yes/no) was also tested and reported. We assessed the assumptions of linear regression, including linearity, homoscedasticity, and multicollinearity, to ensure the validity of our models. All analyses were run on the sample with complete data for all accelerometer metrics, covariates, and physical function outcomes.

### Sensitivity analysis

To assess the robustness of our results, analyses were repeated to assess the impact of slight variations in the analytical sample due to the availability of data for different covariates. These included rerunning analyses involving participants with any combination of the physical function outcomes (rather than just on those with data on all outcomes). In addition, to further assess the potential impact of oversampling of diabetes, we repeated analyses and substituted the binary classification of type 2 diabetes status (yes/no) for a 3-level classification which included a class for pre-diabetes. Finally, we additionally included DHD as an additional predictor to evaluate the potential influence of self-reported diet quality on the association between physical activity and physical function. Analyses were conducted using Stata v17.0 (StataCorp, USA).

## Results

**Fig. 2:**
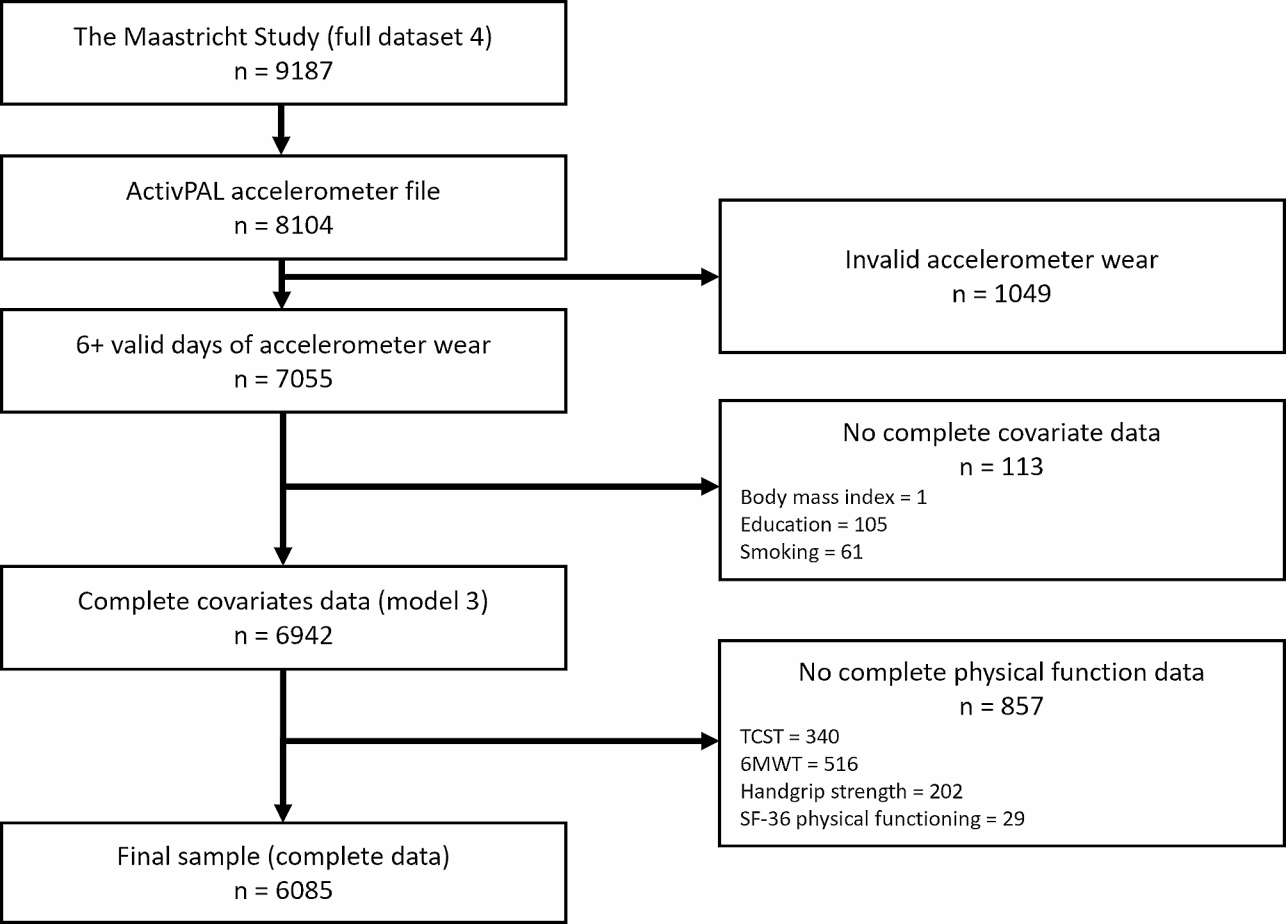
Flow chart of The Maastricht Study participants through our study

A total of 6085 participants, (50.5% female), with a mean (SD) age of 59.6 ± 8.7 years, had 6 (18.8%) or 7 (88.2%) valid days of accelerometer data (with an average waking wear time of 16.4 ± 1.0 h), covariates data, and all physical function outcomes (Fig. 2). Excluded participants were more likely to be overweight, current smokers, have lower education, and poorer performance in physical function tests, except for grip strength. Men had higher grip strength, 6MWT distance, and SF-36pf (all *p*-values < 0.05), but there was no difference between chair rise test time (*p* = 0.56). Participant characteristics are presented in Table 2.

When mutually adjusted for all covariates, there were clear differences in upright event metrics by age, sex, diabetes, education, BMI, smoking status (Additional File 1).

Total step volume was associated with better performance in all three performance-based physical function outcomes (except for grip strength in males), and a higher SF-36pf score for both males and females. The associations in the fully adjusted model are summarised for each physical function outcome below.

**Table 2:** Summary of participant characteristics, upright and stepping event metrics, and physical function outcomes

Participant characteristics	Male (*n* = 3013)	Female (*n* = 3072)	Total (*n* = 6085)
Age	60.7 ± 8.6	58.6 ± 8.7	59.6 ± 8.7
Type 2 diabetes, *n* (%)^a^	812 (27%)	367 (12%)	1179 (24%)
BMI category, *n* (%)			
Healthy (18.5 to 24.9 kg·m^2^)	915 (30%)	1470 (48%)	2,385
Overweight (25 to 29.9 kg·m^2^)	1485 (49%)	1096 (36%)	2,581
Obese (30 to 39.9 kg·m^2^)	588 (20%)	477 (16%)	1,065
Morbidly Obese (≥ 40 kg·m^2^)	25 (1%)	29 (1%)	54
Education level, *n* (%)			
High	1345 (45%)	1109 (36%)	2,454
Medium	805 (27%)	884 (29%)	1,689
Low	863 (29%)	1079 (35%)	1,942
Smoking status, *n* (%)			
Never	1091 (36%)	1325 (43%)	2,416
Former	1527 (51%)	1426 (46%)	2,953
Current	395 (13%)	321 (10%)	716
**Upright and stepping event metrics**			
Daily step count (steps/day)	9457 ± 3759	9747 ± 3395	9604 ± 3582
Daily number of upright events (n/day)	52.2 ± 13.3	52.9 ± 13.0	52.6 ± 13.1
Daily number of stepping events (n/day)	186.7 ± 58.9	209.0 ± 57.1	198.0 ± 59.1
Mean duration of all step events (min/event)	33.3 ± 9.7	30.0 ± 7.6	31.6 ± 8.8
Mean number of steps per all stepping events (n/event)	48.1 ± 18.6	43.2 ± 14.2	45.6 ± 16.7
Step-weighted mean cadence (steps/min)	90.4 ± 9.4	90.7 ± 7.8	90.6 ± 8.6
Duration of all upright events (min)	7.0 ± 2.6	8.0 ± 3.0	7.5 ± 2.9
Proportion of stepping to standing time (%)	35.7 ± 5.8	34.9 ± 5.2	35.3 ± 5.6
Number of steps per upright event (n/event)	189.2 ± 85.4	192.9 ± 79.0	191.1 ± 82.2
Number of stepping events per upright event (n/event)	7.9 ± 2.8	9.2 ± 3.1	8.5 ± 3.0
Upright event burstiness (*B*_n_)	0.28 ± 0.09	0.33 ± 0.08	0.31 ± 0.09
Sedentary event burstiness (*B*_n_)	0.32 ± 0.09	0.32 ± 0.07	0.32 ± 0.08
**Physical function metrics**			
Grip strength (kg)	41.8 ± 8.2	26.0 ± 5.6	33.8 ± 10.6
Six-min walk test (meters)	604.7 ± 82.8	579.4 ± 73.3	591.9 ± 79.2
10x chair stand test (s)	24.7 ± 5.5	24.8 ± 5.7	24.7 ± 5.6
SF-36 Physical functioning score	88.8 ± 14.8	86.5 ± 16.2	87.7 ± 15.6

Mean ± SD or *n* (%)

^a^ Row percentage

### Grip strength

A higher number of stepping events per day was associated with lower grip strength in both males and females. Duration of stepping event was positively associated with grip strength, and number of steps per stepping event was positively associated in females. (Table 3.)

### Timed chair stand test

Upright event metric associations with TCST performance were differentially associated with sex. For males, duration of stepping event and number of steps per stepping event were associated with poorer TCST performance, as was step count within upright events. For females, number of upright events per day and step-weighted average cadence was associated with better TCST performance, as was a higher sedentary burstiness. (Table 4.)

### Six-minute walk test

Sedentary burstiness was associated with better 6MWT test performance in both males and females. Number of steps per stepping event, and step-weighted average cadence were also both associated with better 6MWT in both males and females. Duration of stepping events was positively associated with 6MWT in females only. For both sexes, a higher number of stepping events was associated with poorer performance the 6MWT. (Table 5.)

### SF-36 physical function

A higher upright event burstiness score was associated with a poorer SF-36pf score in males. A higher sedentary burstiness was associated with a better SF-36 score in females. For both males and females, step-weighted average cadence was positively associated with SF-36pf, but to a greater degree in females. Females also has a positive association with duration and number of steps per stepping event and SF-36pf, as was within upright event stepping proportion and step count. (Table 6.)

### Sensitivity analyses

When running analyses on participants without all physical function outcomes, sample sizes increased for all outcomes; handgrip strength (*n* = 6740), TCST (*n* = 6602), 6MWT (*n* = 6426), and SF-36pf (*n* = 6913). With the larger sample sizes, nine of the eighty-eight associations across all upright metrics and physical function outcomes in males and females changed significance. The four of these which became non-significant were sedentary burstiness with 6MWT for males, number of step events with SF-36pf for males, and duration of step events and within upright event step count with SF-36pf for females. When substituting the binary diabetes classification for the WHO classification, which includes pre-diabetes, none of the associations changed significance. These associations are highlighted in Additional File 2. Inclusion of DHD score as an additional predictor yielded negligible changes to the observed study findings. Further, the reduction in sample size (*n* = 5668) due to availability of DHD score precludes definitive conclusions about whether these small changes can be attributed to confounding effects of diet quality per se, or to differences in the analytical sample.

**Table 3:** Associations of upright and stepping event metrics with handgrip strength

	Model 1		Model 2		Model 3	
	Males	Females	Males	Females	Males	Females
	B [95% CI] (*p*-value)	B [95% CI] (*p*-value)	B [95% CI] (*p*-value)	B [95% CI] (*p*-value)	B [95% CI] (*p*-value)	B [95% CI] (*p*-value)
**Daily step count** ^**b**^	0.15 [-0.08,0.38]	0.21 [-0.04,0.46]	0.12 [-0.11,0.36]	**0.27 [0.02,0.52]**	-	-
*(per + 3582 steps)*	(0.19)	(0.094)	(0.294)	(0.037)	-	-
**Upright events**	-0.11 [-0.35,0.13]	-0.15 [-0.39,0.09]	-0.02 [-0.25,0.22]	0.01 [-0.23,0.25]	-0.04 [-0.28,0.20]	0.00 [-0.25,0.24]
*(per + 13.1 n)*	(0.36)	(0.222)	(0.89)	(0.934)	(0.729)	(0.971)
**Stepping events** ^b^	-0.17 [-0.41,0.08]	-0.19 [-0.43,0.06]	-0.17 [-0.42,0.07]	-0.12 [-0.36,0.13]	**-0.45 [-0.73,-0.17]**	**-0.38 [-0.66,-0.10]**
*(per + 59.1 n)*	(0.18)	(0.141)	(0.156)	(0.346)	(0.001)	(0.007)
**Duration of stepping events**	**0.31 [0.09,0.53]**	**0.60 [0.33,0.88]**	**0.29 [0.07,0.50]**	**0.60 [0.32,0.88]**	**0.35 [0.09,0.61]**	**0.67 [0.35,0.99]**
*(per + 8.8 s)*	(0.005)	(< 0.001)	(0.01)	(< 0.001)	(0.007)	(< 0.001)
**Steps per stepping event**	**0.24 [0.02,0.45]**	**0.51 [0.23,0.79]**	0.21 [-0.01,0.42]	**0.51 [0.22,0.79]**	0.22 [-0.03,0.48]	**0.52 [0.20,0.84]**
*(per + 16.7 steps)*	(0.029)	(< 0.001)	(0.057)	(< 0.001)	(0.081)	(0.001)
**Step-weighted cadence**	0.12 [-0.10,0.34]	0.11 [-0.15,0.37]	0.05 [-0.17,0.27]	0.11 [-0.15,0.38]	-0.04 [-0.28,0.20]	0.02 [-0.27,0.30]
*(per + 8.6 steps/min)*	(0.267)	(0.404)	(0.655)	(0.405)	(0.722)	(0.913)
**Duration of upright events**	-0.01 [-0.27,0.24]	-0.04 [-0.26,0.19]	-0.07 [-0.33,0.18]	-0.10 [-0.32,0.13]	-0.16 [-0.43,0.10]	-0.16 [-0.38,0.07]
*(per + 2.9 min)*	(0.911)	(0.746)	(0.577)	(0.398)	(0.233)	(0.176)
**Stepping proportion of upright events**	**0.30 [0.07,0.53]**	0.20 [-0.06,0.45]	**0.23 [0.00,0.46]**	0.13 [-0.12,0.38]	0.18 [-0.05,0.42]	0.09 [-0.17,0.34]
*(per + 5.6%)*	(0.011)	(0.126)	(0.047)	(0.308)	(0.126)	(0.496)
**Step count of upright events**	0.20 [-0.03,0.43]	0.20 [-0.04,0.45]	0.13 [-0.10,0.35]	0.15 [-0.09,0.40]	-0.03 [-0.36,0.29]	0.00 [-0.33,0.33]
*(per + 82.3 steps)*	(0.086)	(0.106)	(0.281)	(0.22)	(0.834)	(0.997)
**Stepping events within upright events**	-0.03 [-0.28,0.23]	-0.11 [-0.34,0.11]	-0.06 [-0.31,0.20]	-0.14 [-0.37,0.08]	-0.15 [-0.42,0.12]	-0.22 [-0.45,0.01]
*(per + 3.0 n)*	(0.845)	(0.322)	(0.671)	(0.217)	(0.266)	(0.065)
**Upright event burstiness**	-0.24 [-0.48,-0.00]	-0.02 [-0.28,0.24]	-0.20 [-0.44,0.04]	0.05 [-0.21,0.31]	-0.22 [-0.46,0.02]	0.04 [-0.22,0.30]
*(per + 0.09)*	(0.05)	(0.882)	(0.1)	(0.697)	(0.075)	(0.75)
**Sedentary event burstiness**	0.07 [-0.15,0.29]	0.24 [-0.02,0.50]	0.08 [-0.14,0.29]	**0.29 [0.03,0.55]**	0.03 [-0.20,0.25]	0.26 [-0.01,0.52]
*(per + 0.08)*	(0.545)	(0.074)	(0.486)	(0.029)	(0.816)	(0.058)

Results are presented as regression coefficient (B) with 95% confidence interval [95% CI] and *p*-value, where the predictor is standardised and the outcome is unstandardised (a one standard deviation increase in the predictor equates to an absolute change in the physical function outcome. Associations were adjusted for the following covariates; Model 1: age, sex, and waking wear time. Model 2: model 1 + type 2 diabetes, education level, body mass index, and smoking status. Model 3: model 2 + average daily step count. ^a^ denotes significant sex interaction (*p* < 0.05) for Model (1) ^b^ denotes significant type 2 diabetes interaction (*p* < 0.05) for Model (2) Bold indicates statistical significance (*p* < 0.05)

**Table 4:** Associations of upright and stepping event metrics with timed chair stand test

	Model 1		Model 2		Model 3	
	Males	Females	Males	Females	Males	Females
	B [95% CI] (*p*-value)	B [95% CI] (*p*-value)	B [95% CI] (*p*-value)	B [95% CI] (*p*-value)	B [95% CI] (*p*-value)	B [95% CI] (*p*-value)
**Daily step count**	**-0.78 [-0.97,-0.60]**	**-1.03 [-1.23,-0.82]**	**-0.51 [-0.70,-0.33]**	**-0.68 [-0.89,-0.48]**	-	-
*(per + 3582 steps)*	(< 0.001)	(< 0.001)	(< 0.001)	(< 0.001)	-	-
**Upright events**	**-0.33 [-0.52,-0.13]**	**-0.43 [-0.62,-0.23]**	**-0.20 [-0.40,-0.01]**	**-0.26 [-0.46,-0.07]**	-0.13 [-0.32,0.06]	**-0.22 [-0.42,-0.02]**
*(per + 13.1 n)*	(0.001)	(< 0.001)	(0.038)	(0.009)	(0.191)	(0.029)
**Stepping events**	**-0.55 [-0.75,-0.35]**	**-0.52 [-0.73,-0.32]**	**-0.46 [-0.65,-0.26]**	**-0.46 [-0.66,-0.26]**	-0.16 [-0.38,0.07]	-0.17 [-0.39,0.05]
*(per + 59.1 n)*	(< 0.001)	(< 0.001)	(< 0.001)	(< 0.001)	(0.174)	(0.138)
**Duration of stepping events** ^**a**^	**-0.38 [-0.56,-0.21]**	**-0.83 [-1.06,-0.60]**	-0.11 [-0.28,0.07]	**-0.37 [-0.60,-0.14]**	**0.33 [0.13,0.54]**	0.13 [-0.13,0.38]
*(per + 8.8 s)*	(< 0.001)	(< 0.001)	(0.244)	(0.001)	(0.001)	(0.337)
**Steps per stepping event** ^**a**^	**-0.42 [-0.60,-0.25]**	**-0.89 [-1.12,-0.67]**	-0.15 [-0.33,0.02]	**-0.43 [-0.66,-0.21]**	**0.25 [0.05,0.45]**	0.03 [-0.22,0.29]
*(per + 16.7 steps)*	(< 0.001)	(< 0.001)	(0.089)	(< 0.001)	(0.016)	(0.791)
**Step-weighted cadence** ^**a b**^	**-0.66 [-0.84,-0.48]**	**-1.02 [-1.24,-0.81]**	**-0.38 [-0.56,-0.20]**	**-0.62 [-0.83,-0.40]**	-0.16 [-0.35,0.04]	**-0.39 [-0.62,-0.16]**
*(per + 8.6 steps/min)*	(< 0.001)	(< 0.001)	(< 0.001)	(< 0.001)	(0.113)	(0.001)
**Duration of upright events**	**-0.22 [-0.43,-0.00]**	-0.03 [-0.22,0.15]	-0.2 [-0.41,0.01]	-0.09 [-0.27,0.09]	0.02 [-0.19,0.24]	0.06 [-0.12,0.24]
*(per + 2.9 min)*	(0.046)	(0.727)	(0.065)	(0.314)	(0.826)	(0.526)
**Stepping proportion of upright events**	**-0.38 [-0.57,-0.20]**	**-0.24 [-0.45,-0.03]**	**-0.33 [-0.52,-0.15]**	-0.16 [-0.36,0.04]	-0.15 [-0.34,0.04]	0.00 [-0.21,0.21]
*(per + 5.6%)*	(< 0.001)	(0.025)	(< 0.001)	(0.116)	(0.123)	(0.999)
**Step count of upright events**	**-0.51 [-0.70,-0.33]**	**-0.58 [-0.78,-0.38]**	**-0.32 [-0.51,-0.14]**	**-0.38 [-0.58,-0.18]**	**0.31 [0.05,0.57]**	0.23 [-0.04,0.49]
*(per + 82.3 steps)*	(< 0.001)	(< 0.001)	(0.001)	(< 0.001)	(0.018)	(0.090)
**Stepping events within upright events**	-0.20 [-0.41,0.02]	-0.01 [-0.20,0.18]	**-0.23 [-0.44,-0.02]**	-0.12 [-0.30,0.07]	-0.01 [-0.22,0.21]	0.06 [-0.12,0.25]
*(per + 3.0 n)*	(0.072)	(0.906)	(0.030)	(0.213)	(0.946)	(0.506)
**Upright event burstiness**	-0.01 [-0.21,0.19]	-0.20 [-0.42,0.01]	0.12 [-0.07,0.31]	-0.07 [-0.28,0.14]	0.17 [-0.02,0.36]	-0.05 [-0.25,0.16]
*(per + 0.09)*	(0.902)	(0.062)	(0.22)	(0.489)	(0.084)	(0.666)
**Sedentary event burstiness**	**-0.31 [-0.49,-0.13]**	**-0.41 [-0.62,-0.19]**	**-0.24 [-0.41,-0.06]**	**-0.35 [-0.56,-0.14]**	-0.06 [-0.24,0.12]	**-0.23 [-0.44,-0.02]**
*(per + 0.08)*	(0.001)	(< 0.001)	(0.009)	(0.001)	(0.510)	(0.035)

Results are presented as regression coefficient (B) with 95% confidence interval [95% CI] and *p*-value, where the predictor is standardised and the outcome is unstandardised (a one standard deviation increase in the predictor equates to an absolute change in the physical function outcome. Associations were adjusted for the following covariates; Model 1: age, sex, and waking wear time. Model 2: model 1 + type 2 diabetes, education level, body mass index, and smoking status. Model 3: model 2 + average daily step count. ^a^ denotes significant sex interaction (*p* < 0.05) for Model (1) ^b^ denotes significant type 2 diabetes interaction (*p* < 0.05) for Model (2) Bold indicates statistical significance (*p* < 0.05)

**Table 5:** Associations of upright and stepping event metrics with six-minute walk test

	Model 1		Model 2		Model 3	
	Males	Females	Males	Females	Males	Females
	B [95% CI] (*p*-value)	B [95% CI] (*p*-value)	B [95% CI] (*p*-value)	B [95% CI] (*p*-value)	B [95% CI] (*p*-value)	B [95% CI] (*p*-value)
**Daily step count**	**19.98 [17.57,22.39]**	**22.33 [19.69,24.98]**	**11.25 [9.04,13.47]**	**12.02 [9.60,14.44]**	-	-
*(per + 3582 steps)*	(< 0.001)	(< 0.001)	(< 0.001)	(< 0.001)	-	-
**Upright events**	**6.06 [3.46,8.67]**	**6.66 [4.00,9.31]**	1.65 [-0.64,3.95]	1.33 [-1.02,3.69]	0.13 [-2.14,2.41]	0.45 [-1.88,2.78]
*(per + 13.1 n)*	(< 0.001)	(< 0.001)	(0.158)	(0.268)	(0.909)	(0.704)
**Stepping events** ^**b**^	**8.54 [5.89,11.20]**	**7.26 [4.56,9.97]**	**4.88 [2.55,7.22]**	**4.65 [2.27,7.02]**	**-3.69 [-6.34,-1.04]**	**-3.49 [-6.14,-0.83]**
*(per + 59.1 n)*	(< 0.001)	(< 0.001)	(< 0.001)	(< 0.001)	(0.006)	(0.01)
**Duration of stepping events** ^**a**^	**15.77 [13.45,18.10]**	**22.23 [19.27,25.18]**	**7.98 [5.88,10.09]**	**10.1 [7.40,12.79]**	2.10 [-0.34,4.54]	**3.44 [0.40,6.47]**
*(per + 8.8 s)*	(< 0.001)	(< 0.001)	(< 0.001)	(< 0.001)	(0.092)	(0.027)
**Steps per stepping event** ^**a**^	**16.46 [14.17,18.74]**	**23.20 [20.24,26.15]**	**8.85 [6.78,10.92]**	**11.00 [8.30,13.70]**	**3.61 [1.21,6.02]**	**4.89 [1.83,7.94]**
*(per + 16.7 steps)*	(< 0.001)	(< 0.001)	(< 0.001)	(< 0.001)	(0.003)	(0.002)
**Step-weighted cadence** ^**a**^	**19.90 [17.58,22.22]**	**23.61 [20.83,26.38]**	**11.83 [9.72,13.94]**	**12.54 [10.00,15.07]**	**8.32 [6.03,10.61]**	**8.92 [6.23,11.61]**
*(per + 8.6 steps/min)*	(< 0.001)	(< 0.001)	(< 0.001)	(< 0.001)	(< 0.001)	(< 0.001)
**Duration of upright events** ^**a**^	**5.08 [2.23,7.94]**	1.21 [-1.24,3.67]	**4.18 [1.69,6.66]**	**2.59 [0.45,4.73]**	-0.10 [-2.64,2.44]	-0.34 [-2.51,1.82]
*(per + 2.9 min)*	(< 0.001)	(0.333)	(0.001)	(0.018)	(0.940)	(0.756)
**Stepping proportion of upright events**	**6.25 [3.72,8.77]**	**5.1 [2.33,7.87]**	**4.77 [2.57,6.97]**	**3.71 [1.30,6.12]**	1.06 [-1.19,3.31]	0.43 [-2.01,2.87]
*(per + 5.6%)*	(< 0.001)	(< 0.001)	(< 0.001)	(0.003)	(0.357)	(0.730)
**Step count of upright events**	**14.32 [11.85,16.79]**	**14.14 [11.49,16.78]**	**8.57 [6.37,10.76]**	**8.52 [6.18,10.86]**	-1.02 [-4.09,2.06]	-0.61 [-3.72,2.50]
*(per + 82.3 steps)*	(< 0.001)	(< 0.001)	(< 0.001)	(< 0.001)	(0.517)	(0.699)
**Stepping events within upright events**	**3.15 [0.30,6.00]**	0.20 [-2.29,2.70]	**3.52 [1.03,6.01]**	**2.60 [0.42,4.78]**	-0.94 [-3.48,1.60]	-0.98 [-3.20,1.24]
*(per + 3.0 n)*	(0.030)	(0.873)	(0.006)	(0.019)	(0.470)	(0.387)
**Upright event burstiness**	**3.30 [0.66,5.94]**	**5.82 [2.95,8.68]**	-1.11 [-3.43,1.20]	1.60 [-0.90,4.11]	-2.07 [-4.35,0.21]	1.05 [-1.42,3.52]
*(per + 0.09)*	(0.014)	(< 0.001)	(0.346)	(0.209)	(0.075)	(0.404)
**Sedentary event burstiness**	**8.16 [5.77,10.54]**	**9.54 [6.65,12.44]**	**5.54 [3.45,7.64]**	**7.57 [5.05,10.10]**	**2.19 [0.04,4.33]**	**5.24 [2.72,7.77]**
*(per + 0.08)*	(< 0.001)	(< 0.001)	(< 0.001)	(< 0.001)	(0.045)	(< 0.001)

Results are presented as regression coefficient (B) with 95% confidence interval [95% CI] and *p*-value, where the predictor is standardised and the outcome is unstandardised (a one standard deviation increase in the predictor equates to an absolute change in the physical function outcome. Associations were adjusted for the following covariates; Model 1: age, sex, and waking wear time. Model 2: model 1 + type 2 diabetes, education level, body mass index, and smoking status. Model 3: model 2 + average daily step count. ^a^ denotes significant sex interaction (*p* < 0.05) for Model (1) ^b^ denotes significant type 2 diabetes interaction (*p* < 0.05) for Model (2) Bold indicates statistical significance (*p* < 0.05)

**Table 6:** Associations of upright and stepping event metrics with SF-36 physical functioning subscale

	Model 1		Model 2		Model 3	
	Males	Females	Males	Females	Males	Females
	B [95% CI] (*p*-value)	B [95% CI] (*p*-value)	B [95% CI] (*p*-value)	B [95% CI] (*p*-value)	B [95% CI] (*p*-value)	B [95% CI] (*p*-value)
**Daily step count** ^**a b**^	**3.14 [2.62,3.67]**	**4.44 [3.87,5.01]**	**1.88 [1.37,2.40]**	**2.87 [2.31,3.43]**	-	-
*(per + 3582 steps)*	(< 0.001)	(< 0.001)	(< 0.001)	(< 0.001)	-	-
**Upright events**	0.55 [-0.01,1.11]	**0.87 [0.30,1.44]**	-0.16 [-0.69,0.37]	-0.04 [-0.58,0.50]	-0.47 [-1.00,0.06]	-0.22 [-0.76,0.32]
*(per + 13.1 n)*	(0.056)	(0.003)	(0.556)	(0.888)	(0.080)	(0.423)
**Stepping events**	**1.45 [0.88,2.02]**	**1.94 [1.36,2.52]**	**0.94 [0.40,1.48]**	**1.52 [0.98,2.07]**	**-0.63 [-1.24,-0.01]**	0.04 [-0.57,0.65]
*(per + 59.1 n)*	(< 0.001)	(< 0.001)	(0.001)	(< 0.001)	(0.046)	(0.899)
**Duration of stepping events** ^**a b**^	**2.52 [2.01,3.02]**	**4.08 [3.44,4.72]**	**1.36 [0.87,1.84]**	**2.23 [1.61,2.86]**	0.14 [-0.43,0.70]	**0.85 [0.15,1.56]**
*(per + 8.8 s)*	(< 0.001)	(< 0.001)	(< 0.001)	(< 0.001)	(0.628)	(0.017)
**Steps per stepping event** ^**a b**^	**2.51 [2.01,3.00]**	**4.29 [3.65,4.93]**	**1.37 [0.89,1.85]**	**2.42 [1.80,3.05]**	0.23 [-0.33,0.78]	**1.09 [0.38,1.79]**
*(per + 16.7 steps)*	(< 0.001)	(< 0.001)	(< 0.001)	(< 0.001)	(0.424)	(0.003)
**Step-weighted cadence** ^**a b**^	**2.86 [2.35,3.36]**	**4.75 [4.14,5.35]**	**1.66 [1.17,2.15]**	**3.05 [2.46,3.64]**	**0.90 [0.37,1.43]**	**2.26 [1.64,2.88]**
*(per + 8.6 steps/min)*	(< 0.001)	(< 0.001)	(< 0.001)	(< 0.001)	(0.001)	(< 0.001)
**Duration of upright events**	**1.08 [0.46,1.69]**	**0.59 [0.06,1.11]**	**0.97 [0.40,1.55]**	**0.84 [0.35,1.34]**	0.14 [-0.44,0.73]	0.27 [-0.23,0.77]
*(per + 2.9 min)*	(0.001)	(0.029)	(0.001)	(0.001)	(0.630)	(0.285)
**Stepping proportion of upright events** ^**a**^	**0.77 [0.23,1.31]**	**1.67 [1.07,2.26]**	**0.58 [0.07,1.08]**	**1.46 [0.91,2.02]**	-0.16 [-0.68,0.36]	**0.81 [0.25,1.38]**
*(per + 5.6%)*	(0.005)	(< 0.001)	(0.026)	(< 0.001)	(0.547)	(0.005)
**Step count of upright events** ^**a b**^	**2.33 [1.80,2.86]**	**3.14 [2.57,3.71]**	**1.51 [1.00,2.01]**	**2.30 [1.76,2.84]**	-0.15 [-0.86,0.57]	**0.73 [0.01,1.45]**
*(per + 82.3 steps)*	(< 0.001)	(< 0.001)	(< 0.001)	(< 0.001)	(0.687)	(0.047)
**Stepping events within upright events**	**0.68 [0.06,1.29]**	0.46 [-0.07,0.99]	**0.78 [0.20,1.35]**	**0.87 [0.37,1.37]**	-0.09 [-0.67,0.50]	0.18 [-0.34,0.69]
*(per + 3.0 n)*	(0.030)	(0.091)	(0.008)	(0.001)	(0.773)	(0.498)
**Upright event burstiness**	-0.14 [-0.71,0.42]	0.42 [-0.19,1.04]	**-0.82 [-1.36,-0.29]**	-0.25 [-0.83,0.33]	**-1.02 [-1.55,-0.49]**	-0.36 [-0.93,0.21]
*(per + 0.09)*	(0.624)	(0.177)	(0.002)	(0.399)	(< 0.001)	(0.215)
**Sedentary event burstiness** ^**a**^	**1.16 [0.65,1.67]**	**2.01 [1.39,2.64]**	**0.77 [0.29,1.26]**	**1.72 [1.14,2.31]**	0.08 [-0.41,0.58]	**1.24 [0.66,1.83]**
*(per + 0.08)*	(< 0.001)	(< 0.001)	(0.002)	(< 0.001)	(0.745)	(< 0.001)

Results are presented as regression coefficient (B) with 95% confidence interval [95% CI] and (*p*-value), where the predictor is standardised and the outcome is unstandardised (a one standard deviation increase in the predictor equates to an absolute change in the physical function outcome. Associations were adjusted for the following covariates; Model 1: age, sex, and waking wear time. Model 2: model 1 + type 2 diabetes, education level, body mass index, and smoking status. Model 3: model 2 + average daily step count. ^a^ denotes significant sex interaction (*p* < 0.05) for Model (1) ^b^ denotes significant type 2 diabetes interaction (*p* < 0.05) for Model (2) Bold indicates statistical significance (*p* < 0.05)

## Discussion

This study aimed to investigate the associations between features of upright and stepping events, including the composition and the temporal distribution, with objective measures of physical function in a large population-based cohort. We observed that greater sedentary burstiness, duration of stepping events, volume of steps per stepping event, and step-weighted cadence were associated with better physical function in one or more of the 6MWT, TCTs, SF-36f, and grip strength outcome, independent of total volume of steps. Number of stepping events was negatively associated with physical function. Upright event composition metrics (within event; duration, proportion of stepping, step count, and number of stepping events) were not associated with physical function outcomes after adjustment for volume. Secondary to our initial focus, it was interesting that there were clear differences in associations between males and females, though the explanation for this is not immediately obvious. Collectively, these findings suggest that some specific dimensions of the pattern in which physical activity is accumulated, are related to physical function, over and above the volume of activity.

These findings contribute to the growing body of research examining the relationship between physical activity patterns and physical function [32]. Our results align with previous studies that have established associations between a higher frequency of short or transient stepping event durations and poorer physical function performance [34, 35]. The mechanism behind these associations is assumed to relate to the capacity of an individual. Higher capacity would likely show a less fragmented physical activity profile, due to the capacity to perform longer bouts of sustained stepping. Our additional examination of the temporal distribution and the composition of upright events provides further insight into how different patterns of physical activity accumulation are related to physical function. In particular, higher sedentary burstiness was associated with better 6MWT performance in both men and women, and better TCST and SF-36pf results particularly in females. Again, we assume these associations relate to capacity, with higher sedentary burstiness meaning greater variation in upright event duration. Conversely, lower sedentary burstiness would be characterised by more uniform upright event durations, which would be shorter due to the finite period of a day, when adjusted for volume. Observed sex differences in many of the associations was interesting, and not immediately understood finding. However, significant sex differences in the upright and stepping metrics were observed here, and in previous research in a mid-life population [43]. 

A potential explanation for positive associations between sedentary burstiness and physical function is that those who undertake a mix of both short and long upright event durations (higher sedentary burstiness) have a higher endurance capacity, compared to females who record mainly short duration upright events. In addition, as the direction of causality is not known due to the cross-sectional design, the associations could also be due to declining physical function decreasing sedentary burstiness. Despite associations with demographic and lifestyle factors [43], the upright event burstiness was not associated with the three performance-based physical function outcomes, and only the SF-36pf in males.

Higher step volume is associated with a range of health outcomes [61], though evidence on the independent effect of step-rate is equivocal [62]. Step-rate has been shown to be associated with a range of health outcomes [63, 64], including the 400-m walk test in older adults; [65] though, conversely, step-rate has not always been shown to be associated with mortality when adjusted for volume [66, 67]. Our results also show that higher step-weighted cadence is associated with better 6MWT performance and SF36-pf score in both males and females, and TCST performance in females, even after adjustment for volume (total daily step count). This could be attributed to our approach to cadence quantification. Unlike previous studies, which primarily relied on step counts above predefined thresholds (e.g., 100 steps/min) and peak cadence metrics (e.g., the 30 highest cadence values per day) [63, 66, 67], or simply the average (unweighted) step-rate over the measurement period [64], our method involves calculating a step-weighted average of all steps. This approach considers the cadence of every step, potentially mitigating the bias associated with fixed thresholds, such as the possibility of someone consistently maintaining a cadence of 90 steps/min without registering any higher-paced stepping, as opposed to individuals who briefly exceed 100 steps/min but predominantly perform lower-paced steps.

### Strengths and limitations

Our study has several strengths, including a large and diverse sample from a population-based cohort and a comprehensive range of physical function outcomes. Previous work has demonstrated the causal relationship between physical activity and physical function [68, 69], however, the cross-sectional nature of our study prevents us from establishing causality. The possibility of reverse causation is present due to the study design, and a degree of bidirectional causation is assumed due to the outcome of choice, poor physical function would be expected to impact physical activity behaviour. Nevertheless, the presence of these associations, irrespective of direction, remains an important finding. Understanding that patterns of physical activity differ for those with poor physical function offers valuable insights for further exploration in this area.

Some limitations of the device-based accelerometer data processing are acknowledged. We used a previously employed, simple, pragmatic method to identify waking wear time [43]. Like other wake/sleep time algorithms, assessment of criterion validity is challenging, and as such there may have been some misclassification, which may have impacted the accuracy of temporal distribution of sedentary and upright burstiness metrics (e.g. if an upright event was registered before the person’s true arise time). In addition, accelerometers are not direct measures of physical activity behaviour but rather a proxy, and proprietary algorithms apply rules, such as minimum resolution of event durations (10 s here), which may result in a level of misclassification potentially underestimating the number of upright events, and therefore the related metrics. Further, here we focussed on metrics describing patterns within days, which prevented examination inter-day variability; future studies might benefit from assessing inter-day variation or considering minimum/maximum daily values for a more nuanced understanding.

The activPAL underestimates slower paced stepping, as the minimum cadence registered is 20 steps/min, potentially leading to an overestimate of stepping cadence [47]. However, the analyses here could not realistically have been achieved with self-reports of physical activity, and adopting an event-based approach allowed for the investigation of the time series of upright and stepping events, as opposed to aggregates or averages over epochs [41]. Adopting an event based approach allowed us to take into account the composition and temporal distribution of upright events, which is not possible when using just aggregate measures of physical activity. These novel metrics of upright and stepping behaviour add to the literature around ‘patterns’ of physical activity and their association with a wide range of health outcomes [32, 34–36, 38]. 

Our study revealed magnitudes of effects that do not reach the clinically meaningful differences established for conventional measures of physical function [70–72]. However, given the novelty of these physical activity metrics (particularly burstiness) and the absence of well-defined standards, we made the deliberate choice to standardize them for analysis. This approach equates a one-standard-deviation change in the predictor to an absolute change in the physical function outcome. Our findings suggest that upright and stepping event measures of physical activity are associated with health outcomes that are not wholly explained by the volume of physical activity undertaken. Accumulation of patterns is different across population sub-groups [43], and having demonstrated these are associated with health outcomes, independent of volume, future work should not ignore how steps are accumulated.

To build upon these findings, prospective population studies are needed with repeat measures, of physical activity and physical function to better understand how trajectories of patterns of physical activity accumulation are associated with changes in physical function. Such studies may also provide insights into what clinically meaningful changes might be. Starting measurements earlier in the life course, prior to loss of function, nay help to determine if changes in patterns of accumulation occur before changes in physical function, or even before declines in physical activity volume occur. If changes in patterns of accumulation, detected before declines in function, predict future declines, intervening earlier in the life course when people still have sufficient function for training programs may be more successful than interventions delivered after significant function is lost.

## Conclusions

In conclusion, our study suggests that patterns of upright and stepping event accumulation, independent of stepping volume, are important consideration in research into physical function. Future research into physical activity and health should examine both physical activity volume and patterns of accumulation to add to our understanding of the benefits of physical activity. Experimental studies are now needed to examine how changing physical activity patterns affects physical function and other health outcomes. A better understanding of how patterns of accumulation are related to health could in the future lead to the refining of public health recommendations, affording individuals greater flexibility in achieving guideline adherence.

### Electronic supplementary material

Below is the link to the electronic supplementary material.

Additional File 1

Additional File 2

## Data Availability

The data of this study derive from The Maastricht Study, but restrictions apply to the availability of these data, which were used under license for the current study. Data are, however, available from the authors upon reasonable request and with permission of The Maastricht Study management team: https://www.demaastrichtstudie.nl/research.
