# Supplementary material for: Cross-sectional associations between patterns and composition of upright and stepping events with physical function: insights from The Maastricht Study
Source: Eur Rev Aging Phys Act. 2024 May 9;21:10. doi: 10.1186/s11556-024-00343-w (PMC11080173; doi:10.1186/s11556-024-00343-w)
Supplement: Supplementary file 1 — Additional File 1 [file 11556_2024_343_MOESM1_ESM.docx]

**Supplementary file 1**

| **Supp.1 Table.1** Associations of participant characteristics with upright and stepping event outcomes. | | | | | | | | |
| --- | --- | --- | --- | --- | --- | --- | --- | --- |
|  |  | **N** | **Upright events (n)** | **Burstiness of upright events** | **Burstiness of sedentary events** | **Stepping events (n)** | **Duration of step events (s)** | **Steps per stepping events (n)** |
| **Sex** (Ref: Male) | | 3016 | Ref. | Ref. | Ref. | Ref. | Ref. | Ref. |
|  | Female | 3072 | 0.19 [-0.46,0.85] | **0.04 [0.04,0.05]** | **-0.01 [-0.01,-0.00]** | **20.18 [17.84,22.52]** | **-3.79 [-4.13,-3.44]** | **-6.01 [-6.67,-5.36]** |
| **Age** (years) | | 6085 | **-0.18 [-0.22,-0.14]** | 0 [-0.00,0.00] | **0 [0.00,0.00]** | **-0.26 [-0.40,-0.12]** | **0.05 [0.03,0.07]** | **0.05 [0.01,0.09]** |
| **Type 2 diabetes**  (Ref: No) | | 4906 | Ref. | Ref. | Ref. | Ref. | Ref. | Ref. |
|  | Yes | 1179 | 0.16 [-0.70,1.02] | **-0.01 [-0.02,-0.01]** | 0 [-0.01,0.00] | -0.48 [-3.55,2.60] | -0.09 [-0.55,0.36] | 0 [-0.86,0.86] |
| **Education** (Ref: Low) | | 1942 | Ref. | Ref. | Ref. | Ref. | Ref. | Ref. |
|  | Med | 1689 | 0.27 [-0.57,1.11] | **-0.01 [-0.01,-0.00]** | **-0.01 [-0.01,-0.00]** | **-3.13 [-6.13,-0.12]** | 0.34 [-0.11,0.78] | 0.62 [-0.22,1.46] |
|  | High | 2454 | -0.42 [-1.20,0.36] | **-0.01 [-0.02,-0.01]** | **-0.02 [-0.02,-0.01]** | **-13.51 [-16.31,-10.72]** | **2.04 [1.63,2.45]** | **4 [3.22,4.79]** |
| **Body mass index**  (Ref: 18.5<25) | | 2385 | Ref. | Ref. | Ref. | Ref. | Ref. | Ref. |
|  | 25<30 | 2581 | **-2.39 [-3.10,-1.67]** | **-0.02 [-0.02,-0.01]** | 0 [-0.00,0.01] | **7.64 [5.08,10.19]** | **-0.76 [-1.14,-0.38]** | **-1.73 [-2.44,-1.01]** |
|  | 30<40 | 1065 | **-5.56 [-6.53,-4.60]** | **-0.03 [-0.04,-0.02]** | 0 [-0.01,0.00] | **5.98 [2.52,9.43]** | **-0.81 [-1.32,-0.30]** | **-1.65 [-2.62,-0.69]** |
|  | ≥40 | 54 | **-9.06 [-12.46,-5.65]** | **-0.03 [-0.06,-0.01]** | -0.01 [-0.03,0.01] | -0.62 [-12.79,11.54] | -0.26 [-2.05,1.54] | -0.99 [-4.39,2.41] |
| **Smoking status**  (Ref: Never) | | 2416 | Ref. | Ref. | Ref. | Ref. | Ref. | Ref. |
|  | Former | 2953 | **0.98 [0.30,1.66]** | **0.01 [0.00,0.01]** | **0 [0.00,0.01]** | 0.58 [-1.85,3.02] | -0.13 [-0.49,0.23] | -0.26 [-0.94,0.42] |
|  | Current | 716 | **4.26 [3.21,5.32]** | 0 [-0.01,0.01] | 0 [-0.01,0.00] | **7.34 [3.57,11.10]** | **-1.18 [-1.74,-0.63]** | **-2.01 [-3.06,-0.96]** |
| Each upright event metric is adjusted for covariates for all covariates in the table, and daily number of steps. | | | | | | | | |

| **Supp.1 Table.2** Associations of participant characteristics with upright and stepping event outcomes. | | | | | | | | |
| --- | --- | --- | --- | --- | --- | --- | --- | --- |
|  |  | **N** | **Daily step count (n)** | **Step-weighted cadence (steps/min)** | **Within upright event composition metrics** | | | |
|  |  |  |  |  | **Duration (min)** | **Stepping proportion (%)** | **Step count (n)** | **Stepping events (n)** |
| **Sex** (Ref: Male) | | 3016 | Ref. | Ref. | Ref. | Ref. | Ref. | Ref. |
|  | Female | 3072 | -115.47 [-293.64,62.71] | **-0.4 [-0.78,-0.02]** | **0.9 [0.76,1.05]** | **-0.75 [-1.03,-0.48]** | **-0.6 [-3.18,1.98]** | **1.21 [1.07,1.36]** |
| **Age** (years) | | 6085 | **-37.00 [-47.67,-26.33]** | **-0.07 [-0.09,-0.05]** | **0.02 [0.01,0.03]** | **-0.06 [-0.08,-0.04]** | **0.65 [0.49,0.80]** | **0.02 [0.01,0.03]** |
| **Type 2 diabetes**  (Ref: No) | | 4906 | Ref. | Ref. | Ref. | Ref. | Ref. | Ref. |
|  | Yes | 1179 | **-1159.98 [-1392.36,-927.59]** | **-0.5 [-0.99,-0.00]** | **-0.24 [-0.43,-0.05]** | 0.23 [-0.13,0.59] | -0.67 [-4.07,2.72] | -0.18 [-0.37,0.01] |
| **Education** (Ref: Low) | | 1942 | Ref. | Ref. | Ref. | Ref. | Ref. | Ref. |
|  | Med | 1689 | -1.80 [-230.34,226.74] | **0.62 [0.13,1.10]** | **-0.22 [-0.40,-0.04]** | 0.16 [-0.19,0.51] | -2.48 [-5.79,0.83] | **-0.36 [-0.55,-0.17]** |
|  | High | 2454 | -52.56 [-265.17,160.04] | **2.13 [1.68,2.58]** | **-0.52 [-0.69,-0.35]** | **0.78 [0.46,1.11]** | -0.31 [-3.39,2.77] | **-0.94 [-1.11,-0.77]** |
| **Body mass index**  (Ref: 18.5<25) | | 2385 | Ref. | Ref. | Ref. | Ref. | Ref. | Ref. |
|  | 25<30 | 2581 | **-790.04 [-983.55,-596.52]** | **-1.15 [-1.56,-0.73]** | **0.4 [0.24,0.55]** | **0.66 [0.36,0.96]** | **9.47 [6.65,12.28]** | **0.56 [0.40,0.72]** |
|  | 30<40 | 1065 | **-1903.04 [-2161.50,-1644.59]** | **-1.38 [-1.94,-0.83]** | **0.79 [0.58,1.00]** | **1.41 [1.00,1.81]** | **20.48 [16.67,24.28]** | **0.84 [0.63,1.06]** |
|  | ≥40 | 54 | **-3593.18 [-4514.43,-2671.94]** | **-2.87 [-4.83,-0.91]** | **1.72 [0.99,2.46]** | 1.19 [-0.23,2.61] | **30.85 [17.45,44.26]** | **1.3 [0.55,2.06]** |
| **Smoking status**  (Ref: Never) | | 2416 | Ref. | Ref. | Ref. | Ref. | Ref. | Ref. |
|  | Former | 2953 | **-250.18 [-435.60,-64.76]** | -0.31 [-0.70,0.09] | **-0.21 [-0.36,-0.06]** | 0.09 [-0.20,0.37] | **-4.01 [-6.69,-1.32]** | -0.09 [-0.24,0.06] |
|  | Current | 716 | **-1468.21 [-1752.47,-1183.96]** | **-1.54 [-2.15,-0.93]** | **-0.43 [-0.65,-0.20]** | 0.04 [-0.40,0.48] | **-11.7 [-15.85,-7.55]** | **-0.33 [-0.56,-0.09]** |
| Each upright event metric is adjusted for covariates for all covariates in the table, and daily number of steps (except daily steps) | | | | | | | | |
