# Supplementary material for: Cross-sectional associations between patterns and composition of upright and stepping events with physical function: insights from The Maastricht Study
Source: Eur Rev Aging Phys Act. 2024 May 9;21:10. doi: 10.1186/s11556-024-00343-w (PMC11080173; doi:10.1186/s11556-024-00343-w)
Supplement: Supplementary file 2 — Additional File 2 [file 11556_2024_343_MOESM2_ESM.docx]

**Supplementary file 2**

| **Supp 2. Table 1.** Sensitivity analyses with full sample size for grip strength (n =6426) and timed chair stand test (n =6602), model 3. | | | | |
| --- | --- | --- | --- | --- |
|  | **Grip strength (kg)** | | **TCST (s)** | |
|  | *Males* | *Females* | *Males* | *Females* |
| **Upright events** | -0.04 [-0.27,0.18] | -0.03 [-0.26,0.20] | -0.13 [-0.32,0.06] | **-0.2 [-0.40,-0.01]** |
| *(per + 13.1 n)* | (0.714) | (0.809) | (0.187) | (0.044) |
| **Burstiness of upright events** | -0.21 [-0.44,0.02] | 0.05 [-0.20,0.30] | 0.18 [-0.02,0.37] | -0.02 [-0.23,0.19] |
| *(per + 0.09)* | (0.072) | (0.678) | (0.077) | (0.857) |
| **Burstiness of sedentary events** | 0.14 [-0.07,0.35] | 0.25 [-0.00,0.50] | -0.16 [-0.34,0.02] | **-0.25 [-0.47,-0.04]** |
| *(per + 0.08)* | (0.198) | (0.052) | (0.085) | (0.022) |
| **Stepping events** | **-0.38 [-0.64,-0.11]** | **-0.37 [-0.64,-0.11]** | -0.18 [-0.40,0.05] | **-0.24 [-0.46,-0.01]** |
| *(per + 59.1 n)* | (0.005) | (0.006) | (0.124) | (0.041) |
| **Duration of stepping events** | **0.41 [0.17,0.65]** | **0.61 [0.30,0.92]** | **0.29 [0.09,0.50]** | 0.06 [-0.21,0.32] |
| *(per + 8.8 sec)* | (0.001) | (<0.001) | (0.005) | (0.675) |
| **Steps per stepping event** | **0.28 [0.04,0.52]** | **0.46 [0.15,0.77]** | **0.24 [0.03,0.44]** | -0.02 [-0.29,0.24] |
| *(per + 16.7 steps)* | (0.023) | (0.004) | (0.023) | (0.868) |
| **Step-weighted cadence** | 0.05 [-0.17,0.28] | 0 [-0.27,0.27] | -0.15 [-0.35,0.04] | **-0.46 [-0.69,-0.24]** |
| *(per + 8.6 steps/min)* | (0.642) | (0.977) | (0.125) | (<0.001) |
| **Duration of upright events** | -0.13 [-0.38,0.12] | -0.11 [-0.33,0.10] | 0.03 [-0.18,0.24] | -0.01 [-0.19,0.17] |
| *(per + 2.9 min)* | (0.316) | (0.303) | (0.78) | (0.924) |
| **Stepping proportion of upright events** | **0.3 [0.08,0.52]** | 0.02 [-0.22,0.26] | -0.18 [-0.37,0.01] | -0.01 [-0.22,0.19] |
| *(per + 5.6 %)* | (0.008) | (0.883) | (0.062) | (0.908) |
| **Step count of upright events** | 0.1 [-0.21,0.41] | 0.02 [-0.30,0.34] | **0.29 [0.03,0.55]** | 0.14 [-0.13,0.41] |
| *(per + 82.3 steps)* | (0.525) | (0.898) | (0.031) | (0.303) |
| **Stepping events within upright events** | -0.09 [-0.34,0.16] | -0.19 [-0.41,0.04] | -0.04 [-0.25,0.18] | 0 [-0.19,0.19] |
| *(per + 3.0 n)* | (0.481) | (0.1) | (0.729) | (0.998) |
| Model 3 adjusted for age, sex, waking wear time, type 2 diabetes, education level, body mass index, smoking status, and average daily step count. Bold indicates statistical significance p < 0.05. Green indicates coefficient became significant with sensitivity analyses. Red indicates coefficient became non-significant with sensitivity analyses. | | | | |

| **Supp 2. Table 2.** Sensitivity analyses with full sample size for six-minute walk test (n =6426) and SF-36 physical functioning (n =6913), model 3. | | | | |
| --- | --- | --- | --- | --- |
|  | **6MWT (m)** | | **SF-36 physical functioning** | |
|  | *Males* | *Females* | *Males* | *Females* |
| **Upright events** | 0.2 [-2.04,2.45] | 0.35 [-1.96,2.66] | -0.45 [-0.99,0.09] | -0.04 [-0.60,0.52] |
| *(per + 13.1 n)* | (0.859) | (0.763) | (0.103) | (0.89) |
| **Burstiness of upright events** | **-2.91 [-5.17,-0.65]** | 1 [-1.45,3.45] | **-1.04 [-1.59,-0.48]** | -0.55 [-1.15,0.05] |
| *(per + 0.09)* | (0.012) | (0.422) | (<0.001) | (0.071) |
| **Burstiness of sedentary events** | 2.11 [-0.01,4.24] | **5.5 [2.99,8.01]** | **0.58 [0.07,1.09]** | **1.57 [0.96,2.18]** |
| *(per + 0.08)* | (0.052) | (<0.001) | (0.026) | (<0.001) |
| **Stepping events** | **-3.08 [-5.70,-0.45]** | **-3 [-5.64,-0.35]** | -0.07 [-0.71,0.57] | 0.44 [-0.20,1.09] |
| *(per + 59.1 n)* | (0.022) | (0.026) | (0.829) | (0.178) |
| **Duration of stepping events** | 1.75 [-0.69,4.19] | **3.26 [0.26,6.26]** | 0.28 [-0.30,0.87] | 0.68 [-0.05,1.42] |
| *(per + 8.8 sec)* | (0.159) | (0.033) | (0.34) | (0.07) |
| **Steps per stepping event** | **3.07 [0.67,5.48]** | **4.6 [1.58,7.62]** | 0.39 [-0.19,0.96] | **0.93 [0.18,1.67]** |
| *(per + 16.7 steps)* | (0.012) | (0.003) | (0.192) | (0.015) |
| **Step-weighted cadence** | **8.1 [5.83,10.37]** | **9.39 [6.71,12.06]** | **1.6 [1.06,2.15]** | **2.56 [1.92,3.21]** |
| *(per + 8.6 steps/min)* | (<0.001) | (<0.001) | (<0.001) | (<0.001) |
| **Duration of upright events** | -0.3 [-2.82,2.22] | -0.45 [-2.60,1.70] | 0.23 [-0.37,0.84] | 0.15 [-0.37,0.67] |
| *(per + 2.9 min)* | (0.817) | (0.68) | (0.448) | (0.581) |
| **Stepping proportion of upright events** | 1.29 [-0.94,3.52] | 1.55 [-0.87,3.97] | 0.27 [-0.27,0.80] | **1.23 [0.65,1.81]** |
| *(per + 5.6 %)* | (0.256) | (0.208) | (0.327) | (<0.001) |
| **Step count of upright events** | -0.96 [-4.04,2.11] | -0.4 [-3.49,2.69] | 0.15 [-0.60,0.89] | 0.7 [-0.06,1.46] |
| *(per + 82.3 steps)* | (0.539) | (0.8) | (0.701) | (0.07) |
| **Stepping events within upright events** | -0.7 [-3.23,1.83] | -0.78 [-3.00,1.43] | 0.25 [-0.35,0.86] | 0.28 [-0.26,0.82] |
| *(per + 3.0 n)* | (0.587) | (0.488) | (0.411) | (0.312) |
| Model 3 adjusted for age, sex, waking wear time, type 2 diabetes, education level, body mass index, smoking status, and average daily step count. Bold indicates statistical significance p < 0.05. Green indicates coefficient became significant with sensitivity analyses. Red indicates coefficient became non-significant with sensitivity analyses. | | | | |

| **Supp 2. Table 3.** Sensitivity analyses substituting binary type 2 classification for WHO classification with pre-diabetes. Model 3 (n =6085). | | | | |
| --- | --- | --- | --- | --- |
|  | **Grip strength (kg)** | | **TCST (s)** | |
|  | *Males* | *Females* | *Males* | *Females* |
| **Upright events** | -0.04 [-0.28,0.20] | 0 [-0.25,0.24] | -0.12 [-0.32,0.07] | **-0.22 [-0.41,-0.02]** |
| *(per + 13.1 n)* | (0.725) | (0.989) | (0.202) | (0.03) |
| **Burstiness of upright events** | -0.22 [-0.46,0.02] | 0.04 [-0.22,0.30] | 0.17 [-0.03,0.36] | -0.05 [-0.25,0.16] |
| *(per + 0.09)* | (0.072) | (0.766) | (0.089) | (0.67) |
| **Burstiness of sedentary events** | 0.03 [-0.19,0.26] | 0.25 [-0.01,0.52] | -0.07 [-0.25,0.11] | **-0.23 [-0.44,-0.02]** |
| *(per + 0.08)* | (0.789) | (0.06) | (0.469) | (0.035) |
| **Stepping events** | **-0.45 [-0.73,-0.18]** | **-0.38 [-0.66,-0.10]** | -0.15 [-0.38,0.07] | -0.17 [-0.39,0.06] |
| *(per + 59.1 n)* | (0.001) | (0.007) | (0.184) | (0.144) |
| **Duration of stepping events** | **0.35 [0.10,0.61]** | **0.67 [0.35,0.99]** | **0.33 [0.13,0.54]** | 0.12 [-0.13,0.38] |
| *(per + 8.8 sec)* | (0.007) | (<0.001) | (0.002) | (0.349) |
| **Steps per stepping event** | 0.22 [-0.03,0.48] | **0.52 [0.20,0.84]** | **0.25 [0.04,0.45]** | 0.03 [-0.23,0.29] |
| *(per + 16.7 steps)* | (0.08) | (0.001) | (0.017) | (0.81) |
| **Step-weighted cadence** | -0.05 [-0.29,0.20] | 0.01 [-0.27,0.30] | -0.16 [-0.35,0.04] | **-0.39 [-0.62,-0.16]** |
| *(per + 8.6 steps/min)* | (0.708) | (0.933) | (0.11) | (0.001) |
| **Duration of upright events** | -0.16 [-0.43,0.10] | -0.16 [-0.38,0.07] | 0.02 [-0.19,0.24] | 0.06 [-0.12,0.24] |
| *(per + 2.9 min)* | (0.225) | (0.172) | (0.836) | (0.528) |
| **Stepping proportion of upright events** | 0.18 [-0.05,0.42] | 0.09 [-0.17,0.34] | -0.15 [-0.34,0.04] | 0 [-0.20,0.21] |
| *(per + 5.6 %)* | (0.127) | (0.5) | (0.133) | (0.991) |
| **Step count of upright events** | -0.04 [-0.36,0.29] | 0 [-0.33,0.32] | **0.31 [0.05,0.57]** | 0.23 [-0.04,0.49] |
| *(per + 82.3 steps)* | (0.83) | (0.995) | (0.019) | (0.093) |
| **Stepping events within upright events** | -0.15 [-0.42,0.11] | -0.22 [-0.45,0.01] | -0.01 [-0.22,0.21] | 0.06 [-0.12,0.25] |
| *(per + 3.0 n)* | (0.257) | (0.065) | (0.955) | (0.505) |
| Model 3 adjusted for age, sex, waking wear time, type 2 diabetes, education level, body mass index, smoking status, and average daily step count. Bold indicates statistical significance p < 0.05. Green indicates coefficient became significant with sensitivity analyses. Red indicates coefficient became non-significant with sensitivity analyses. | | | | |

| **Supp 2. Table 4.** Sensitivity analyses substituting binary type 2 classification for WHO classification with pre-diabetes. Model 3 (n =6085). | | | | |
| --- | --- | --- | --- | --- |
|  | **6MWT (m)** | | **SF-36 physical functioning** | |
|  | *Males* | *Females* | *Males* | *Females* |
| **Upright events** | 0.12 [-2.15,2.39] | 0.5 [-1.82,2.83] | -0.48 [-1.00,0.05] | -0.22 [-0.76,0.31] |
| *(per + 13.1 n)* | (0.919) | (0.671) | (0.075) | (0.414) |
| **Burstiness of upright events** | -2.11 [-4.39,0.17] | 1 [-1.47,3.46] | **-1.01 [-1.54,-0.49]** | -0.36 [-0.93,0.21] |
| *(per + 0.09)* | (0.069) | (0.427) | (<0.001) | (0.214) |
| **Burstiness of sedentary events** | **2.27 [0.13,4.41]** | **5.21 [2.69,7.73]** | 0.09 [-0.40,0.59] | **1.24 [0.66,1.83]** |
| *(per + 0.08)* | (0.037) | (<0.001) | (0.708) | (<0.001) |
| **Stepping events** | **-3.74 [-6.39,-1.09]** | **-3.46 [-6.12,-0.81]** | **-0.63 [-1.25,-0.02]** | 0.03 [-0.58,0.65] |
| *(per + 59.1 n)* | (0.006) | (0.01) | (0.043) | (0.912) |
| **Duration of stepping events** | 2.12 [-0.32,4.56] | **3.44 [0.40,6.47]** | 0.14 [-0.42,0.71] | **0.86 [0.16,1.56]** |
| *(per + 8.8 sec)* | (0.088) | (0.026) | (0.617) | (0.016) |
| **Steps per stepping event** | **3.62 [1.22,6.02]** | **4.9 [1.85,7.95]** | 0.23 [-0.32,0.79] | **1.09 [0.39,1.80]** |
| *(per + 16.7 steps)* | (0.003) | (0.002) | (0.412) | (0.002) |
| **Step-weighted cadence** | **8.28 [5.99,10.56]** | **8.85 [6.16,11.54]** | **0.9 [0.37,1.43]** | **2.26 [1.64,2.89]** |
| *(per + 8.6 steps/min)* | (<0.001) | (<0.001) | (0.001) | (<0.001) |
| **Duration of upright events** | -0.15 [-2.68,2.39] | -0.37 [-2.52,1.79] | 0.15 [-0.44,0.73] | 0.27 [-0.23,0.77] |
| *(per + 2.9 min)* | (0.909) | (0.74) | (0.622) | (0.283) |
| **Stepping proportion of upright events** | 1.04 [-1.21,3.28] | 0.41 [-2.02,2.85] | -0.17 [-0.69,0.35] | **0.81 [0.25,1.37]** |
| *(per + 5.6 %)* | (0.365) | (0.741) | (0.527) | (0.005) |
| **Step count of upright events** | -1.03 [-4.10,2.04] | -0.62 [-3.73,2.49] | -0.14 [-0.85,0.57] | **0.73 [0.01,1.45]** |
| *(per + 82.3 steps)* | (0.511) | (0.695) | (0.699) | (0.046) |
| **Stepping events within upright events** | -0.99 [-3.53,1.55] | -0.99 [-3.20,1.23] | -0.09 [-0.68,0.50] | 0.18 [-0.34,0.69] |
| *(per + 3.0 n)* | (0.444) | (0.382) | (0.767) | (0.498) |
| Model 3 adjusted for age, sex, waking wear time, type 2 diabetes, education level, body mass index, smoking status, and average daily step count. Bold indicates statistical significance p < 0.05. Green indicates coefficient became significant with sensitivity analyses. Red indicates coefficient became non-significant with sensitivity analyses. | | | | |
